# Models to Predict Development or Recurence of Hepatocellular Carcinoma (HCC) in Patients with Advanced Hepatic Fibrosis

**DOI:** 10.1007/s11894-022-00835-8

**Published:** 2022-02-10

**Authors:** Mignote Yilma, Varun Saxena, Neil Mehta

**Affiliations:** 1 Department of Surgery, University of California, San Francisco, CA USA; 2 Department of Gastroenterology and Transplant Hepatology, Kaiser Permanente South San Francisco, San Francisco, CA USA; 3 Division of Gastroenterology, University of California, San Francisco, CA USA

**Keywords:** HCC, De novo development, Recurrence

## Abstract

**Purpose of Review:**

Hepatocellular carcinoma (HCC) is the third leading cause of cancer-related death in the United States (U.S.).^1^ The purpose of this review is to highlight published models that predict development of HCC and estimate risk of HCC recurrence after treatments.

**Recent Findings:**

There have been several models created for both de novo HCC and HCC recurrence, with the more recent models using a combination of age, sex, decompensation, and laboratory values (platelet count, albumin, bilirubin), and liver disease etiology to predict both 5 and 10-year HCC incidence. For chronic hepatitis C, sustained virologic response has been a useful component of understanding HCC risk reduction. BMI and diabetes have been utilized in non-alcoholic fatty liver disease (NAFLD) models to predict HCC risk. For HCC recurrence after treatment (for both surgical resection and liver transplant), tumor size and number, vascular invasion, alpha-fetoprotein (AFP) and neutrophil to lymphocyte ratio (NLR) are all components of HCC recurrence risk models.

**Summary:**

Although numerous HCC risk prediction models have been established over the last several years, challenges remain including how to best incorporate these models into clinical practice, improve surveillance for NAFLD-HCC development, and determine timing and duration of post-resection recurrence surveillance.

## Introduction

Hepatocellular carcinoma (HCC) is the fifth most common cause of cancer and the third leading cause of cancer-related death in the United States (U.S.). [1] The incidence of HCC has increased more than threefold, which coincides with rising prevalence of non-alcoholic fatty liver disease (NAFLD). [1, 2] While cirrhosis is considered to be a precursor for most HCC cases, HCC can occur in the absence of cirrhosis [3] with major risk factors for HCC development including viral hepatitis, alcohol-associated liver disease (ALD), and NASH. [4] In addition, advanced liver fibrosis and cirrhosis are major risk factors for HCC, with 70–90% of all detected HCC occurring in patients with chronic liver disease or cirrhosis. [5, 5]

In this review, we first discuss published models created to predict development of HCC followed by a review of models created to estimate risk of HCC recurrence after treatments. Risk stratification strives for a more personalized approach to HCC surveillance by ideally identifying low-risk individuals, who may be able to avoid surveillance or require a less intensive approach, as well as identifying high-risk individuals, who may benefit from more aggressive HCC surveillance.

## Models for De Novo HCC Development

### Cirrhosis with Mixed Etiology of Liver Disease

There have been several models developed to predict risk of HCC development in the setting of chronic liver disease (Table 1). The ADRESS-HCC model was based on a cohort of 17,124 cirrhotic patients from a national liver transplant waitlist database. In this cohort, the overall incidence of HCC was 2.9 per 100 person-years and multivariate analysis showed that age, sex, race, diabetes, etiology of cirrhosis, and severity of liver disease (Child–Pugh score) were statistically associated with HCC development. Based on this model, an ADRESS-HCC score model was developed to predict the 1-year risk of developing HCC with a score ≥ 4.67 able to identify patients with HCC risk of ≥ 1.5% per year. When the ADRESS-HCC model was applied to patients with preserved hepatic function (i.e. Child–Pugh A), the median ADRESS-HCC score was 4.96 with a corresponding 1-year median HCC development risk of 2%. [6]

**Table 1:** HCC occurrence risk stratification models

Author, year	Components	Derivation Population	Validation Cohort/Clinical Utility
Cirrhosis with Mixed Etiology of Liver Disease			
Flemming, 2014 [6]	Age, sex, race, diabetes, etiology of cirrhosis, and Child–Pugh score	17,124 mixed etiology cirrhotic patients to predict 1-year risk of HCC C-index: 0.70	Validation cohort of 17,808 patients, with c-index of 0.69 ADRESS-HCC score $\ge$ 4.67 was able to identify patients with HCC risk of $\ge$ 1.5% per year
Sharma, 2017 [7]	Age, gender, etiology, platelet count	2,079 mixed etiology cirrhotic patients to predict 5 and 10-year HCC incidence C-index 0.76 with platelet as continuous variable and 0.75 when platelet as categorical variable, and 0.74 when excluding F3	External validation cohort of 1,144 with c-index 0.76 when including F3 patients, and 0.74 when excluding F3 patients
Chronic Hepatitis C (HCV)			
Kanwal, 2017 [8•]	Age, Race, Cirrhosis, alcohol use, sustained virologic response (SVR)	19,518 HCV patients with $\ge$ 6 months completion of DAAs and SVR, with HCC developing in 183 patients	SVR after DAA therapy reduces HCC risk in HCV cirrhosis patients by 72% (HCC annual incidence after SVR 0.90 compared to 3.45 without SVR)
Chronic Hepatitis B (HBV)			
Yuen, 2009 [9]	GAG-HCC: sex, age, HBV DNA, cirrhosis	820 HBV patients with 5- and 10-year prevalence of HCC of 4.4% and 6.3%, respectively	No validation cohort
Wong, 2010 [10]	CU-HCC: age, albumin, bilirubin, HBV DNA, cirrhosis	1,005 HBV patients with 10.4% incidence of HCC at 10 years follow-up	External validation of 424 patients with 10.6% of HCC incidence at 10-year follow-up Medium and high-risk groups had hazards ratio for HCC of 12.8 and 14.6, respectively
Yang, 2011 [11]	REACH-B: age, sex, ALT, HBeAg status, HBV DNA	3,584 HBV patients with incidence of HCC of 0–23.6% at 3 years, 0–47% at 5 years, and 0–81.6% at 10 years	Validation cohort of 1,505 patients, predict with model showing AUROC of 0.81 for HCC risk at 3 years, 0.80 at 5 years, and 0.77 at 10 years 1,228 non-cirrhotic validation patients with AUROC of 0.90 for risk at 3 years, 0.78 at 5 years, and 0.81 at 10 years
Wong, 2014 [12]	LSM-HCC Liver: age, albumin, HBV DNA, liver stiffness measurement	1,035 HBV patients with 3- and 5-year cumulative incidence of HCC 2.3% and 3.3%, respectively	Internal validation of 520 patients with 3- and 5-year cumulative incidence of HCC 1.5% and 2.9%, respectively
Papatheodoridis, 2016 [13]	PAGE-B: platelet count, age, sex	1,325 patients with CHB and post-anti-viral therapy for over 12 months with 5-year cumulative HCC incidence. PAGE-B $\le$ 9, 10–17, $\ge$ 18 with 5-year rates of 0%, 4%, and 7% C-index of 0.82	Internal and external validation of 490 patients. C-index of 0.81 and 0.82, respectively PAGE-B $\le$ 9, 10–17, $\ge$ 18 with 5-year cumulative HCC incidence of 0%, 4%, 16%
Kim, 2018 [14]	m-PAGE-B: age, sex, platelet count, albumin	2,001 Asian patients with HBV receiving anti-viral therapy with five-year cumulative HCC incidence of 6.6%	Internal and external validation (n = 1000) five-year cumulative HCC incidence of 7.2% Five-year cumulative incidence of HCC with m-PAGE-B score $\le$ 8, 9–13, and $\ge$ 13 were 1.9%, 6.5%, and 18.2%, respectively
Hsu, 2018 [15]	CAMD: cirrhosis, age, sex, diabetes	23,851 HBV patients receiving antiviral therapy with 3-year HCC incidence of 3.56% C-index of 0.83, 0.82, and 0.82 at first, second, and third years	Internal validation cohort of 19,321 with c-index 0.74, 0.75, and 0.75 at first three years; and 0.76 at fourth and fifth years CAMD score < 8 (low risk) 0.7% three-year cumulative incidence, 8–13 (intermediate risk) 3.4% incidence, and > 13 (high risk) 9.2%
Lee, 2020 [16]	CAMPAS: cirrhosis on ultrasound, age, sex, platelet count, albumin, liver stiffness measurement	1,511 HBV patients with virological response with 0.43%, 0.85% and 1.40% for HCC development at 3, 5, and 7 years for CAMPAS score of < 75, and 3.3%, 6.4%, and 10.5% for score > 161 C-index of 0.87	Internal and external validation (n = 252). CAMPAS score 75–161 (intermediate) and score > 161 (high-risk) were more likely to develop HCC than low-risk group C-index of 0.87
Cirrhosis due to non-alcoholic fatty liver disease (NAFLD) and alcohol-associated liver disease (ALD)			
Ioannou, 2019 [17]	Age, sex, BMI, diabetes, platelet count, serum albumin and serum AST/$\sqrt{ALT}$ ratio	7,068 NAFLD-cirrhotic and 16,175 ALD-cirrhotic pts to predict HCC risk C-index 0.76 in ALD-cirrhosis, 0.75 for NAFLD-cirrhosis, and 0.76 for combined	Internal validation cohorts with c-index of 0.74 for ALD-cirrhosis, 0.72 for NAFLD-cirrhosis, and 0.73 for combined etiology

The Toronto HCC risk index (THRI) was developed using a cohort of 2,079 cirrhotic patients with the most common etiology being chronic hepatitis C (HCV), followed by chronic hepatitis B (HBV), ALD, and NAFLD. 10-year incidence of HCC in HCV was 23% in HBV and 21% in HCV (though this risk decreased to 7% in those who achieved sustained virologic response (SVR)), 18% in ALD and 13% in NAFLD. THRI was derived using age, etiology of liver disease, gender, and platelet count. This score was able to stratify 10-year cumulative HCC incidence into low (< 3%), intermediate (10%), and high (32%) risk categories with c-statistic ranging from 0.74–0.76. [7]

### Chronic Hepatitis C (HCV)

Chronic infection with HCV is a leading risk factor for HCC, with annual risk of 2–4% in patients with cirrhosis. [18] In a cohort of 214 HCV RNA seropositive patients, 32% developed HCC with an annual incidence rate of 3.9%. [4] Among a cohort of 1500 Veterans Affairs (VA) HCC patients, 68% had HCV, with ~ 90% having confirmed cirrhosis at the time of HCC diagnosis. [18] Direct acting-antivirals (DAAs) have led to 90% of patients with compensated cirrhosis and 80% of decompensated cirrhotic patients being able to achieve SVR. [19] Among patients with cirrhosis who have achieved SVR, there is no difference in HCC incidence or HCC-free survival between DAAs and IFN. [20] In a VA based study of patients with known HCV infection that were receiving IFN (58%), DAA (35%) or a combination of both (7.3%), the incidence of HCC was highest in patients with cirrhosis and treatment failure (3.25 per 100 patient years) and lowest in patients with no cirrhosis and SVR (0.24). [21].

In a prospective study of patients with HCV receiving DAAs who had achieved SVR (97% in F3 fibrosis, 93% in Child–Pugh A, and 80% in Child–Pugh B), one-year incidence of HCC was 0.5% in F3 fibrosis, 1.5% in CP-A, and 3.6% in CP-B patients. Multivariate analysis showed that AST to Platelet Index Ratio (APRI) score > 2.5 and HBV coinfection were independent risk factors for the development of HCC in patients with cirrhosis. Of the patients that developed HCC, 60% had achieved SVR; the more aggressive patterns of HCC were present in 55% of patients without SVR compared to 12% of patients with SVR. [19] In another large cohort study of VA patients with HCV receiving DAAs (Table 1), the annual incidence of HCC in those with SVR was 0.90 per 100 patient years (in comparison to 3.45 per patient years in those without SVR) with SVR associated with an overall 72% risk reduction of HCC [8•]. In patients with DAA induced SVR, the risk of HCC in patients with cirrhosis was nearly 5 times higher than for those without cirrhosis, and the risk for HCC in patients with alcohol use was 1.5 times higher than for those without alcohol use. Patients with fibrosis-4 index (FIB-4) > 3.25 were 6 times more likely to develop HCC than those with FIB-4 < 1.4 with additional risk factors including diabetes and alcohol with a 2- and threefold increase, respectively [8•].

### Chronic Hepatitis B (HBV)

Among patients with chronic hepatitis B (HBV), the risk of developing HCC is highly variable, but presence of cirrhosis is the strongest risk factor. [22] The risk for HCC in Asian HBV carriers without cirrhosis is 0.3 – 0.6% per year while it is 3–8% per year in those with cirrhosis. [22] Numerous risk scores have been developed in a variety of HBV populations (Table 1). Earlier predictions models had HBV DNA as a component of their scoring. GAG-HCC was based on 820 HBV patients; its predictors included sex, age, HBV DNA, and cirrhosis. [9] Wong et al. were able to categorize patients as medium or high-risk based on age, albumin, bilirubin, HBV DNA and cirrhosis. [10] REACH-B was able to predict HCC risk for both cirrhotic and non-cirrhotic patients. [11] LSM-HCC, a liver stiffness based prediction model is able to predict cumulative incidence of HCC at 3- and 5-year. [12].

The PAGE-B (platelet, age, sex) based on HBV patients who have received anti-viral therapy for $\ge$ 12 months had 5-year cumulative incidence of HCC ranging from 0 to 16%. [13] Kim et al. applied this scoring method to Asian patients with HBV, m-PAGE-B, which had five-year cumulative incidence of HCC ranging from < 2% up to nearly 20% for those with the highest risk score. [14] The CAMD score (cirrhosis, age, male sex and diabetes) based on a large cohort of HBV patients receiving antiviral therapy had a five-year cumulative incidence of HCC ranging from < 1% up to nearly 10% based on CAMD score [15] with Lee et al. developing a similar CAMPAS HCC risk model. [16].

### Non-Alcoholic Fatty Liver Disease (NAFLD) and Alcohol-associated Liver Disease (ALD)

NAFLD has recently become the most common cause of chronic liver disease in U.S. [1, 4, 5] Non-alcoholic steatohepatitis (NASH), which develops in 10–20% of NAFLD patients, accounts for up to 40% of cryptogenic HCC cases. [23, 24] NAFLD has a prevalence of 30% in adult population, and 70–90% in those with obesity and type 2 diabetes. [25, 26] Fatty liver without cirrhosis has been recognized to have a significant role in the risk of HCC development presumably due to underlying metabolic risk factors including diabetes and obesity. [27] For example, in a multicenter study, 7% of patients developed HCC in the absence of cirrhosis during a 1-year period [28] and NAFLD-HCC patients are 5 times more likely to have HCC in the absence of cirrhosis than HCV-HCC patients. [29]

Patients with HCC secondary to NAFLD appear less likely to be diagnosed by surveillance compared to patients with HCC secondary to viral hepatitis. In one study, only 43% of NAFLD associated HCC patients underwent HCC surveillance within 3 years of their HCC diagnosis in comparison to 87% in HCV associated HCC and 60% in alcohol associated HCC. [23] Part of the problem leading to under-surveillance is that NAFLD-HCC patients often have no evidence of underlying cirrhosis. Among HCC patients with metabolic syndrome (excluding HCV, HBV and alcohol abuse), only 67% had confirmed cirrhosis at time of HCC diagnosis. [4] Additionally, in NAFLD-HCC patients without cirrhosis, liver fibrosis was absent in > 50% with < 20% having F3/advanced fibrosis. [30] Thus, NAFLD-HCC patients often are not receiving surveillance, are diagnosed at a later tumor stage and in this setting, are more likely to be ineligible for HCC-specific treatment. [23].

Ioannou et al. studied VA cirrhotic patients due to NAFLD and ALD and showed that patients with NAFLD-cirrhosis were older, had higher BMI, and were more likely to have diabetes. During a mean follow-up of 3.7 years, 5.5% of patients with cirrhosis developed HCC. with similar annual incidence of HCC (ALD 1.4%, NAFLD 1.6%). The annual incidence was greater in those with FIB-4 > 3.25 at 2.7% than those with FIB-4 < 3.25 at 0.7%. Increasing BMI and diabetes were strong predictors in ALD-cirrhosis but not in NAFLD-cirrhosis. The model to predict HCC incidence in NAFLD or ALD cirrhotic patients were developed separately and included seven predictors: age, sex, BMI, diabetes, platelet count, serum albumin and serum AST/$\sqrt{\mathrm{ALT}}$ ratio. Of these, four (age, platelet count, serum AST/$\sqrt{\mathrm{ALT}}$ ratio, and albumin) accounted for majority of the prediction model with a c-statistic of 0.72–0.74 in the validation dataset (Table 1) [17•].

## Models to Predict HCC Recurrence

Of the multiple HCC staging systems which stratify patients to determine appropriate treatment options, the Barcelona Clinic Liver Cancer (BCLC) classification [31] is the most commonly utilized. For patients with advanced stage HCC (e.g. extra-hepatic disease or main portal vein tumor thrombus), nearly all staging classifications and society guidelines recommend pursuing systemic therapy (which is not considered curative and thus not discussed in this review). On the other hand, for HCC patients with BCLC stage 0 (single lesion < 2 cm) or stage A (single lesion or 2–3 lesions, each < 3 cm), resection, ablation, and liver transplantation (LT) are recommended by most professional society guidelines. [32–34] These potentially curative treatments offer 5-year survival above 60–70% [35–40] with LT associated with the best survival and lowest risk of recurrence. [37, 40–42] While HCC recurrence after potentially curative treatment is not uncommon, long-term survival can be achieved with early recurrence detection [43, 44] and so ongoing HCC surveillance is recommended. Surveillance can be optimally performed by estimating individual recurrence risk through established risk scores that typically include type of tumor treatment, pathological analysis (in the case of resection and LT), and biomarkers (e.g. AFP).

### Recurrence after Surgical Resection

While resection for early-stage HCC is increasingly being performed due to rising HCC incidence and organ shortages, numerous studies have shown very high 5-year recurrence rates after resection approaching 50–70% with almost tenfold higher odds of recurrence compared to LT. [40, 42]Early detection of post-resection recurrence is critical given the possibility of salvage transplant for those detected within Milan criteria. [40] In terms of established risk factors, presence of cirrhosis in the background liver significantly increases recurrence risk [45] with a recent multi-center matched case–control series finding that post-resection recurrence occurs in > 70% of patients with cirrhosis compared to < 40% with histologically normal liver parenchyma. [46] The importance of tumor size and number as a predictor of post-resection outcome was demonstrated in a large multi-national study [46–48] of HCC patients treated with LT (n = 1218) or resection (n = 2068) to determine the likelihood of statistical cure. Overall survival rates after resection dropped dramatically with increasing tumor burden, ranging from 60% with a single small < 3 cm lesion to 10% for patients with either > 3 tumors or a single > 8 cm tumor.

Several models incorporating tumor burden and cirrhosis/liver function have recently been developed to predict post-resection recurrence (Table 2). In a multi-national study, Chan et al [49•] was able to stratify early recurrence within 2 years of resection into three risk strata with a model incorporating male sex, increasing tumor size and number, albumin-bilirubin grade, AFP, and microvascular invasion. Patients in the highest risk strata had 2-year recurrence free survival of only 20% compared to 65% in the lowest risk group. A multi-center study from China [48] found that these same variables (except for AFP) were also associated with late recurrence (occurring more than 2 years after resection). Finally, a post-operative nomogram developed in Singapore [47] found that these same variables (along with symptoms at presentation and surgical margins) predicted the development of any recurrence (i.e. early and late) following curative HCC resection.

**Table 2:** HCC recurrence risk stratification models

Author year	Components	Derivation population	Validation cohort/Clinical Utility
Surgical resection			
Ang, 2015 [47]	Cirrhosis, Child–Pugh, symptoms, Tumor size, multifocality, vascular invasion, surgical margin AFP	405 patients undergoing first-line curative surgery, predicted any HCC recurrence C-index: 0.69	No validation cohort Model provides superior estimation of clinical net benefit for recurrence threshold > 40% vs common staging systems (e.g. BCLC)
Xu, 2019 [50]	Male sex, cirrhosis, tumor size > 5 cm, multiple/satellite tumors, vascular invasion	734 patients, predicted late HCC recurrence > 2 years after resection	No validation cohort or model performance reported Regular surveillance for recurrence was an independent predictor of overall survival for patients with late recurrence
Chan, 2018 [49]	Male sex, albumin-bilirubin, grade, tumor size and number, microvascular invasion, AFP	451 patients, predicted early recurrence < 2 years after resection C-index: 0.73	Validation cohort: 3,322 patients from US, Japan, Italy, and China with C-index: 0.62–0.72
Liver Transplantation			
Halazun, 2017 [51]	Tumor size and number Vascular invasion Stage of tumor differentiation AFP, NLR	339 HCC patients undergoing LT C-index: 0.91	No validation cohort Model stratifies LT recipients into very high risk with 20% 5-year survival compared to > 90% in lowest risk HCC patients
Agopian, 2015 [52]	Tumor size and number Vascular invasion Stage of tumor differentiation AFP, NLR, cholesterol	865 HCC patients undergoing LT C-index: 0.85	No validation cohort Clinicopathologic prognostic nomogram accurately predicts post-LT HCC recurrence risk at 1-, 3-, and 5-years
Mehta, 2017 [53] 2018 [54]	Tumor size and number Vascular invasion AFP	1060 HCC patients undergoing LT C-statistic: 0.77	Validation cohort: UNOS national database (n = 3392) with C-index 0.75 Model stratifies post-LT recurrence risk at 75% within 5-years for highest risk (RETREAT ≥ 5) compared to 3% in lowest risk (RETREAT 0)

### Post-Transplant Recurrence Models

LT for HCC patients within Milan criteria (including after successful down-staging) offers excellent long-term outcome, though post-LT recurrence occurs in up to 15% [37, 40–42] and remains the most common cause of death in this population with a median survival of ~ 1 year from recurrence. There has been a recent push to incorporate markers of tumor biology into selection criteria, rather than simply focusing on tumor size and number. Examples of pre-LT criteria to minimize post-LT recurrence include AFP < 20 ng/mL, decreasing AFP slope, AFP-L3 < 10–15%, DCP < 7.5 ng/mL, and FDG-negative PET scan. [55] However, once a patient undergoes LT, the explant provides a wealth of objective data to improve HCC recurrence risk prediction, which can be used to guide surveillance strategies and potentially tailor immunosuppression.

Similar to resection, several post-LT recurrence risk prediction models have recently been developed (Table 2). The combo-MORAL score [51] includes preoperative NLR ≥ 5, maximum AFP > 200 ng/mL along with tumor differentiation, vascular invasion, and tumor number and size and has excellent recurrence prediction (AUROC 0.91) though has not yet been validated. In a similar large single center experience, Agopian et al [52] developed a prognostic nomogram based on nearly 900 HCC LT recipients that includes many of these same variables along with total cholesterol. Finally, the RETREAT score, [53] which has been validated nationally [53] (including in patients requiring tumor down-staging [54]) incorporates AFP at LT, vascular invasion, and the sum of the largest viable tumor diameter (in cm) and number of viable tumors on explant. RETREAT stratifies 5-year recurrence risk from < 3% in patients without viable tumor or vascular invasion on explant and AFP ≤ 20 ng/mL (i.e. RETREAT score of 0) up to 75% in the highest risk patients (RETREAT ≥ 5). Additionally, 3-year post-LT survival decreases with increasing RETREAT score: 91% for a score of 0, 80% for a score of 3, and 58% for a score ≥ 5. [54] Patients with a RETREAT score of 0 have such low recurrence risk that they likely do not benefit from surveillance whereas RETREAT-based surveillance has been proposed for all others with increasing frequency and length of surveillance for those with higher RETREAT scores. [56].

### Recurrence after Local–Regional Therapy

Tumor ablation is gaining acceptance as an alternative first-line treatment to resection for small solitary tumors given lower morbidity and similar long-term outcomes compared to resection. [57] Importantly, recurrence rates after ablation are directly related to tumor diameter. For single tumors ≤ 2 cm, ablation has been proposed as the treatment of choice given complete response rates > 90% [36] with > 70% 5-year overall survival [58] and low rates of recurrence beyond Milan [58] compared to response rates of only 50% for lesions > 3 cm with recurrence approaching 80%. [59].

As opposed to resection and LT, models to predict recurrence after local–regional treatments (LRT) including Y90 radioembolization, transarterial chemoembolization (TACE), and stereotactic body radiation therapy (SBRT) are not well established. This is in part because these modalities are used to treat a very heterogenous group of tumors (e.g. BCLC stages A, B, and locally advanced C) and since tumor recurrence even after initial complete response is quite common. Y90 results in similar overall survival compared to TACE but longer time to progression for early or intermediate stage HCC. [60] Data on external stereotactic body radiotherapy (SBRT) as primary treatment for HCC are emerging, though often when other LRT have failed or are no longer feasible. SBRT delivers focused radiation under image guidance sparing large portions of the liver while providing ablative potential within the tumor, achieving local HCC control rates around 90% and similar survival compared to TACE and radiofrequency ablation. [50, 61, 62] Similar to that seen in the resection and LT literature, factors influencing post-LRT recurrence and overall survival include tumor number and size, AFP, liver dysfunction (e.g. bilirubin, albumin, Child–Pugh score), and vascular invasion. [63] Additionally, response to LRT (e.g. mRECIST) is an important marker of tumor biology with progressive disease associated with particularly poor outcome. [63–65].

## Conclusions

Numerous HCC risk prediction models have been established over the past 10 years. For de novo HCC development, multiple models specific to liver disease etiology and presence of cirrhosis can provide individualized risk of HCC development. However, challenges remain including how best to incorporate these risk-based models into clinical practice to tailor HCC surveillance regimens and how to improve surveillance for NAFLD-HCC development, especially in the absence of NAFLD-cirrhosis. Once HCC develops and treatment is undertaken, recurrence risk models account for treatment type with highest recurrence risk seen with local–regional therapy followed by resection with the lowest risk observed with LT. After surgical treatment, all recurrence risk models incorporate markers of tumor biology (e.g. AFP, vascular invasion, tumor size/number) with cirrhosis an additional powerful marker of recurrence after resection. While risk-based surveillance regimens have been proposed after surgical HCC treatment and early detection of post-resection recurrence can allow for salvage transplant, studies are still needed to determine optimal timing and duration as well as the clinical impact of surveillance after surgical HCC treatment.
